# The National and Regional Prevalence Rates of Disability, Type, of Disability and Severity in Saudi Arabia—Analysis of 2016 Demographic Survey Data

**DOI:** 10.3390/ijerph15030419

**Published:** 2018-02-28

**Authors:** Saad M. Bindawas, Vishal Vennu

**Affiliations:** Department of Rehabilitation Sciences, College of Applied Medical Sciences, King Saud University, P.O. Box 10219, Riyadh-11433, Saudi Arabia; vvennu@ksu.edu.sa

**Keywords:** Saudi Arabia, disability, prevalence, national study

## Abstract

The prevalence of disability varies between countries ranging from less than 1% to up to 30% in some countries, thus, the estimated global disability prevalence is about 15%. However, it is unknown what the current estimate of disability and its types and severity are in Saudi Arabia. Thus, the objective of this study is to estimate national and regional prevalence rates of any disability, types of disability, and their severity among Saudi populations. Data on disability status were extracted from the national demographic survey conducted in 2016 as reported by the General Authority for Statistics, Saudi Arabia (N = 20,064,970). Prevalence rates per a population of 100,000 of any disability, type of disability, and its severity were calculated at the national level and in all 13 regions. Out of 20,064,970 Saudi citizens surveyed, 667,280 citizens reported disabilities, accounting for a prevalence rate of 3326 per a population of 100,000 (3.3%). Individuals aged 60 years and above (11,014) and males (3818) had a higher prevalence rate of disability compared with females (2813). The Tabuk region has the highest rate of reported disability, at 4.3%. The prevalence rates of extreme disabilities in mobility and sight were higher in Madinah (57,343) and Northern border (41,236) regions, respectively. In Saudi Arabia, more than half a million Saudi citizens (1 out of every 30 individuals) reported the presence of disability during the year 2016. A higher prevalence rate of disability was seen among those aged 60 years and above, and males. Targeted efforts are required at the national and regional levels to expand and improve rehabilitation and social services for all people with disabilities.

## 1. Introduction

According to World Health Organization, the International Classification of Functioning, Disability, and Health (ICF) outlinesdisability as an umbrella term, confining impairment, activity, and participation limitations [1]. In other words, disability is part of the human condition that will temporarily or permanently impair almost everyone at some point in life, and those who survive to old age will experience increasing difficulties in functioning [2]. Substantial evidence suggests that disabilities related to aging and many health conditions can be reduced and functioning improved with rehabilitation [3]. However, there is still no global consensuson the operational measures of disability and data on the need for rehabilitation services. This knowledge limitation, including cultural variations in the perception of disabilities and the demand for rehabilitation services, can be estimated using data on the prevalence of disability or disability-specific surveys.

A primary concern of measuring disability prevalence for countries are examining the level of functioning in a population, designing service delivery, and determiningthe equalization of opportunity [4]. Moreover, the measure gives robust evidence, helpingto make well-informed decisions about disability policies and programmes [2]. These strategies and plans help to understand the numbers of people with disabilities, their circumstances, and barriers. Evidently, the global disability prevalence was estimated at 15% [2], yet it varies significantly between countries ranging from less than 1% to up to 30% in some other countries [5]. The difference in the prevalence of disability between nations can be explained by several factors, such as differing definitions of disability, different methodology, and the difference in study design [6,7,8]. Additionally, personal factors, such as age, gender, education, and employment status, and environmental factors, such as climate, topography or building design, attitudes, institutions, and laws differ between the countries and communities, as well as the relevance of associated health conditions [2]. Thus, estimateddisability prevalence rates are not reasonable and are challenging to compare internationally.

In Saudi Arabia, disability is a significant social and economic problem [9]. The source of national data, and data collections approaches of any disability are little, primarily on aging populations [10]. However, insufficient published studies addressed the epidemiology of disability in Saudi Arabia [9,10,11]. Each type of disability and its severity has particular health, educational, rehabilitation, social, and support needs to help design specific services. The usefulness of such data is limited in Saudi Arabia as the prevalence rates are not suggestive of the overall level of disability [2].

Therefore, this study was set out to estimate the national and regional prevalence rates of any disability, types of disability, and its severity among Saudi populations. This investigation will strengthen our understanding of the national and local prevalence and be compared to the global disability prevalence studies.

## 2. Materials and Methods 

**Figure 1:**
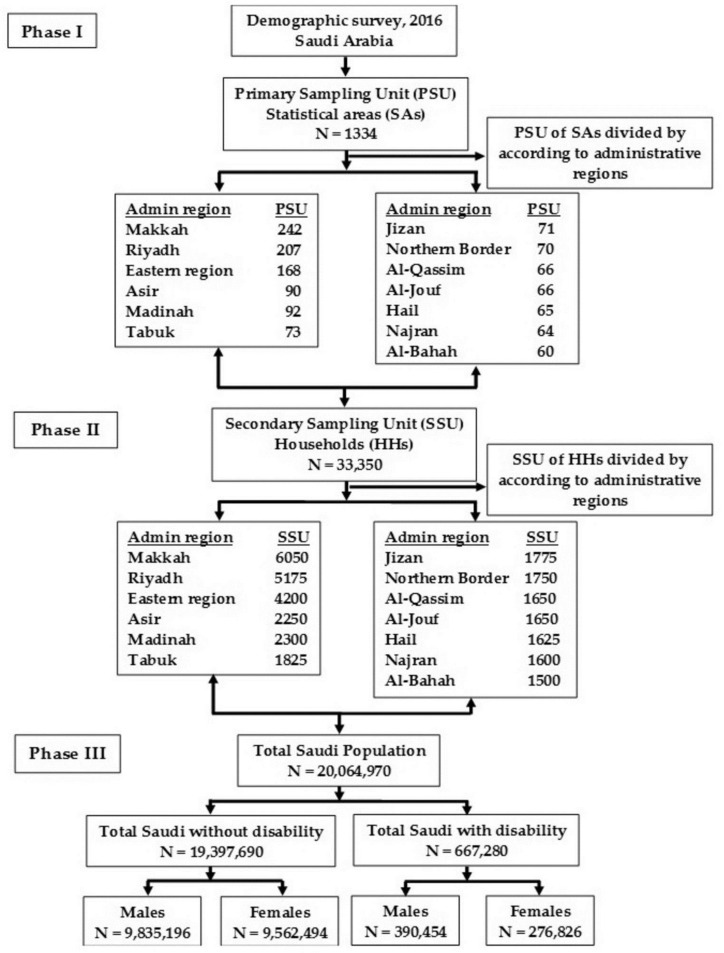
The flow of demographic survey sample selection in 2016.

The detailed flow of the demographic survey and the sample selection aresummarized in Figure 1.

The survey covers all administrative regions in Saudi Arabia (N = 13) conducted by the General Authority for Statistics (GASTAT) using the general population and housing census framework. The survey was carried out in each administrative region to collect the data from each selected household (N = 33,350) between 29 April 2016, and 7 June 2016. The definition of the variables used in the 2016 survey are presented in Table 1.

Field researchers from GASTAT were trained to interview the head of the household and record all the data electronically using the hand devices “tablet” in the research form [12]. The form met all international standards, recommendations, and was evaluated by experts in the field of demography and many other ministries, such as the Ministry of Interior, Ministry of Economy and Planning, and Ministry of Health. The form consists of many questions in the Arabic language, including two questions about difficulty, type of difficulty, and its degree, which were based on the six questions of the Washington Group (WG) on Disability Statistics [13].

In this descriptive study, data on disability status of Saudi citizens (N=20,064,970) were extracted from the 2016 demographic survey, as reported by the GASTAT. According to the 2016 survey, the following questions about disability were used: “Does the person suffer a difficulty in mobility, hearing, remembering, self-care, and speech?” Having a disability was defined if someone answered “yes” to the above question. The type of disability and its severity was assessed using the following question among persons with disability: “Type of difficulty in senses (such as eyesight, hearing, mobility, memory, self-care, speech and communication) and its degree”. This question has three levels of response, designed to capture the full spectrum of difficulty (light, severe, and acute/I cannot). These two questions in the survey are not the same as the six questions used in the WG form. However, similar questions were used in several studies from different countries, including some from the Middle East region [14].

The prevalence rates per 100,000 populations of any disability, type of disability, and its severity were calculated at the national level and each administrative region in Saudi Arabia. The numerator was number of persons with a disability, type of disability, severity, sex, and age groups, while the denominators were taken from the 2016 survey. All the analyses were performed using a Microsoft Excel spreadsheet, 2010 version 14.0 running on Windows 7 (Microsoft Corporation, Redmond, WA, USA).

## 3. Results

The prevalence rates of any disability in 2016 among Saudi citizens by basic demographic variables are shown in Table 2. Out of 20,064,970 Saudi populations, 667,280 citizens reported any disability in 2016 accounting for a prevalence rate of 3326 per 100,000 populations (3.3%) in Saudi Arabia. 

**Figure 2:**
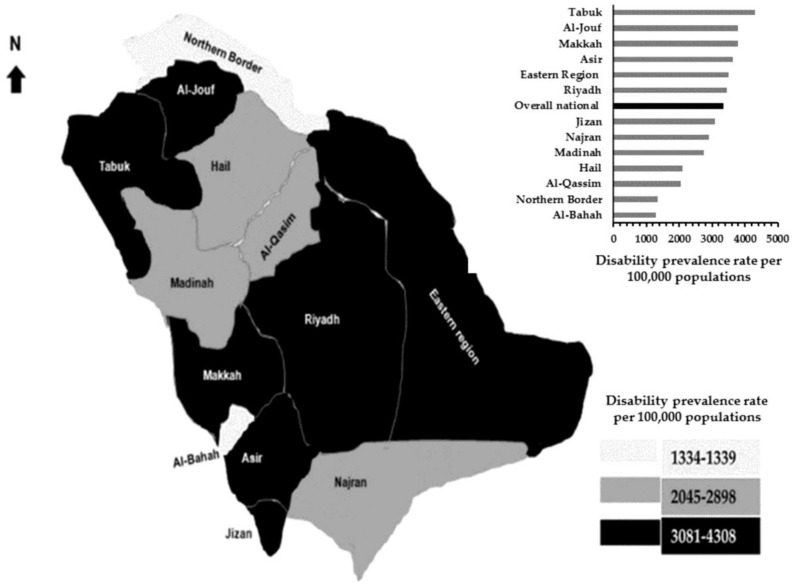
The national and regional disability prevalence rates per 100,000 populations in 2016.

**Figure 3:**
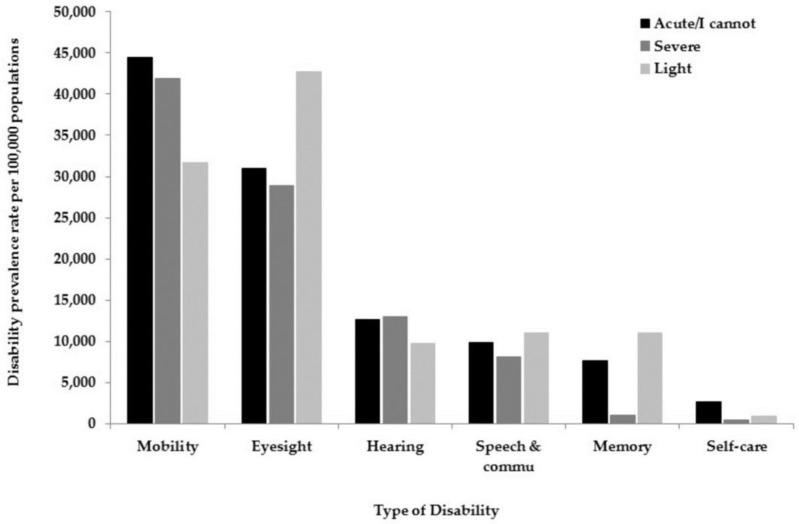
Disability prevalence rates per 100,000 populations in 2016 according to the type of disability and severity.

The national and regional prevalence rates per 100,000 populations of any disability in 2016 are summarized in Figure 2. Out of 13 administrative regions, six regions (Tabuk, Al-Jouf, Makkah, Asir, Eastern Region, and Riyadh) prevalence rates of any disability, ranging from 3081 to 4308 and above the national average rate (3326). Among remain seven regions, two regions (Northern Border and Al-Bahah), ranging from 1339 to 1304 and lower than any region in the country. The five remaining regions range from 2045 to 2898 (Jizan, Najran, Madinah, Hail, and Al-Qassim). The type of disability and its severity prevalence rates per 100,000 populations in 2016 areillustrated in Figure 3.

The prevalence rates per 100,000 populations in 2016 in all administrative regions according to the type of disability and its severity are presented in Table 3. Disability in mobility was reported as an extreme difficulty type in Madinah (57,343), followed by Jizan (53,653) and Al-Bahah (51,186). The disability associated with eyesight was reported as an extreme difficulty in the Northern Border (41,236) and Hail (40,016). The extreme difficulty in the hearing was reported in Madinah (17,445), while communication was reported in Tabuk (20,331) and Al-Qassim (21,131).

## 4. Discussion

The present study was set out to estimate the national and regional prevalence rates of any disability, types of disability, and its severity among Saudi populations. The outcomes of this study show that 1 in every 30 citizens reported any disability. A higher prevalence rate of any disability was seen among older Saudis (1 in every 9) and males (1 in every 26) compared to females (1 in every 35). The prevalence rate of any disability, disabilities in mobility (1 in every 2) and eyesight(1 in every 3) types with an extreme difficulty were higher in Tabuk (1 in every 23), Madinah, and the Northern Border (each 1 in every 2), respectively, than any other regions.

The survey approaches vary from censuses to measuring disability, and the use of these methods to data collection in the same country may report different rates of disability [16]. Censuses in Saudi Arabia are conducted every 10 years, which covers each household in each administrative region. Most questions in censuses cover socioeconomic data and few disability-relevant questions. While dedicated surveys provide more abundant information on disability characteristics that are relevant to policy-makers, such as the type of disability, the degree of its severity, and the use of and need for services [14]. Accordingly, the survey in 2016 used a composite questionnaire listing a set of difficulties, which differ from the six individual questions of the WG [13,14]. The WG applies an ICF-based approach to disability and follows the principles and practices of national statistical agencies as defined by the United Nations Statistical Commission. It covers six functional domains: seeing, hearing, mobility, cognition, self-care, and communication.

Countries that collect disability data through censuses tend to report low disability prevalence rates. For example, an analysis of 2011 census data used to estimate the prevalence of disability among older adults in India [17] showed that 1 in every 20 Indian citizens aged 60 years and above reported any disability. This is lower compared to other developing countries, such as Jamaica (1 in every 12 persons), Pakistan, and Bangladesh (each 1 in every 6 persons) [18]. On the contrary, nations that collect their data through surveys tend to report higher disability prevalence rates. For example, the Department of Health and Human Services in the United States issued guidelines for defining and managing data on disability status using a standard set of questions through a survey in 2013 [19]. The findings from that survey reported that approximately 1 in every 5 adults reported any disability in 2013. Additionally, it reported that disability in mobility was the most frequently reported type (13%) and varied across states. Previous studies have shown that different questionnaires used to measure disability based on various approaches to the disability phenomenon yield varied estimates of prevalence rates [14,20].

It is interesting to record that the current study found that the overall prevalence rate of any disability in Saudi Arabia is lower than the world disability prevalence [21]. In the United States, for example, overall, 35.5% of the older adults aged 65 years and above reported any disability in 2013 [19]. The results of the earlier study also estimated that disability prevalence in all 54 countries is 39.4% for older individuals aged 65 years and above [5]. This is high compared to our findings of disability prevalence (11%) of older adults aged 60 years and above in Saud Arabia. Furthermore, the current study findings demonstrated that any disability rate was higher in male than in female Saudi citizens. This resultdiffers from the analysis across the 54 countries [5]. That study showed that females in all age groups were found to have a higher disability prevalence than males. More research is required on the level of sex determinants of disability prevalence. However, the variation of prevalence rates for disability is not strictly comparable owing to the differences in the definition used in the present study.

Findings in this study are consistent with the earlier studies [17,19]. For example, our result shows disability in mobility was the most frequently reported type than any other types of disability in Saudi Arabia, which is similar to the United States and India [17,19]. In India, mobility and seeing were the most frequently reported disabilities than any other types [17]. Furthermore, the disability associated with eyesight was also reported as an extreme difficulty in Saudi Arabia. This finding was similar to the study from Tamil Nadu, India reported the difficulty in mobility, followed by seeing [22]. Moreover, disability prevalence rates, type of disability and its severity varies in Saudi Arabia across all thirteen regions, which were homogeneous to previous findings [17,19,22]. However, none of the studies reported the degree of severity of any disability.

The findings highlight the need for developing a national policy addressing disability in Saudi Arabia as its prevalence is expected to increase with growing Saudi populations in the next decades. Additionally, there is a need for more rehabilitation services, as was reported in a previous call to action paper for people with disabilities and functional limitation, especially after stroke [23]. It can also help plan the need for medical and social services and may be useful for setting appropriate priorities for rehabilitation hospitals and centers [24]. Most of the data on disability status and unmet need for rehabilitation services are derived from the surveys on a specific population [2,5,6,7,13,15,19]. Therefore, there is also a need to address the challenges in establishing a national integrated information system that use ICF as a framework in collecting accurate, comparable, relevant, and accessible disability data. This system could facilitate the universal access to services and benefits, provides better awareness in legislation and rights of persons with disabilities.

The strengths of the present study, the large number of respondents provides one of the largest samples about disability in Saudi Arabia using the most recent demographic survey, 2016 data. In this survey, field researchers were trained by the GASTAT staff to interview the household head and to systematically collect the data about disability and other topics. The current study findings may provide the basic knowledge about disability for the Saudi Vision 2030 toward a vibrant society.

This method of analysis has some limitations. First, these results need to be interpreted with caution because of thedescriptive nature of the study and the questions used to collect the data on disability would not be the best to verify disability [20]. An examination of various survey questions about communication difficulty from different countries and languages, such as Canada, France, the Netherlands, South Africa, and the United States reveals limitations in their approaches and recommended better inclusive instruments to accurately capture this difficulty in future national surveys [25]. Finally, all the data in the survey were collected as self-reported and, therefore, might be subject to recall and social bias. However, self-reporting is the most commonly used method for assessing disability for similar purposes [19]. It is also recommended that anew Arabic WG six questions survey be used, after establishing the validity and reliability of the Arabic-modified WG in Saudi Arabia 

Many of the barriers persons with any disability face are avoidable, and the disadvantages associated with disability can be overcome. Implementing the following recommendations requires action towards policy development and providing equal opportunities for each person with a disability. National disability policy and plans of action at all levels should be adopted, together with a broad range of stakeholders, to set out a comprehensive long-term vision for improving the well-being of persons with disabilities. Additionally, efficient and effective rehabilitation services are needed to improve the function and independence of persons with disabilities to participate in the social and cultural lives of their communities.

## 5. Conclusions

More than half a million Saudi citizens (1 in every 30 persons) reported any disability in 2016. A higher prevalence rate of any disability was seen among persons aged 60 years and above, and males. Among all regions, Tabuk, Madinah, and the Northern Border were most frequently reported any disability, disability in mobility and eyesight types with extreme difficulty.Future national surveys should use the six questions of WG to ensure accurate estimation ofdisability prevalence, and related factors to fill the knowledge gaps about disability issues, policy, programs, and allocating resources.

## Figures and Tables

**Table 1:** Definition of the variables used in the demographic survey of 2016 in Saudi Arabia.

Variables	Definition
Population	All Saudi individuals, living in the Kingdom at the time of the survey [15].
Survey population	Member of the households selected as a sample who are residing the house and collectively named statistical households, including domestic workers and drivers who live in the same house [15].
Administrative region	Saudi Arabia comprises thirteen administrativeregions (Riyadh, Makkah, Madinah, Al-Qassim, Eastern Region, Asir, Tabuk, Northern Borders, Jizan, Najran, Al-Bahah, Hail, and Al-Jawf). Each one is supervised by a governmental organ affiliated to the Ministry of the Interior. In each region, there is a metropolis where the regions headquarter is based [15].
Household	A person or a group of persons with or without a relationship binding them to one anotherwho share a residence [15]. The household includes: The Saudi nationals who are habitually residing with the same family, but were not available for temporarily traveling abroad when the study was underway. For example, people in business, tourists, patients, and students overseas for medication and education, respectively. Family members who were absent during the data collection process to attend shift nights. For example, guards, physicians, nurses, fisherman, airport staff. Servants, drivers, and the like who are members of the same household. Family members who are on a trip in the Kingdom.
Head of the family	The person considered by the family members as its chief member.Usually, he is responsible for taking decisions on family affairs, and his age must not be below 15 years old. If the family is composed of children and their mother, and they are cared for by kin who does not live with them, such a relationship shall not be considered the head of the family, nor shall he be recorded as one of its members. In this case, the mother shall be found as the head of the family [15].
Age	According to similar international studies, the age is divided into three groups: 0–19 years. 20–59 years. 60 years and above.
Disability	It is an inability or permanent disability that limits the activity and mobility of a person who loses, suffers deviation of a physiological or neurological organ, or a function of an element or limb. In other words, the person experiences shortage or inefficiency in doing activities than a healthy man can do [13].
Person with disability	A person who suffers disability caused by genetic and environmental factors which resulted in a physical or mental impairment that makes it challenging to successfully carry out business or physical and mental activities which might be carried out by healthy persons [13].

**Table 2:** Disability prevalence rates in 2016 by basic demographic variables in Saudi Arabia.

Demographic Characteristics	Total Population N = 20,064,970	Persons with Disability N = 667,280 (3.3%)	Prevalence Rate Per 100,000 of Any Disability
**Age**			
0–19 years	7,849,953	209,574 (2.7%)	2670
20–59 years	10,905,304	313,457 (2.9%)	2874
60 years and above	1,309,713	144,249 (11%)	11,014
**Gender**			
Male	10,225,650	390,454 (3.8%)	3818
Female	9,839,320	276,826 (2.8%)	2813
**Administrative regions**			
Tabuk	710,699	30,617 (4.3%)	4308
Al-Jouf	373,662	14,146 (3.8%)	3786
Makkah	4,440,571	168,096 (3.8%)	3785
Asir	1,719,950	62,624 (3.6%)	3641
Eastern Region	3,087,687	108,267 (3.5%)	3506
Riyadh	4,579,570	157,409 (3.4%)	3437
Jizan	1,187,284	36,582 (3.1%)	3081
Najran	430,711	12,483 (2.9%)	2898
Madinah	1,353,102	37,018 (2.7%)	2736
Hail	529,012	11,044 (2.1%)	2088
Al-Qassim	991,032	20,266 (2.0%)	2045
Northern Border	285,486	3824 (1.3%)	1339
Al-Bahah	376,204	4904 (1.3%)	1304

**Table 3:** Disability prevalence rates per 100,000 populations in 2016 in all 13 administrative regions according to type of disability and severity.

Disability & Severity	Tabuk	Al-Jouf	Makkah	Asir	Eastern Region	Riyadh	Jizan	Najran	Madinah	Hail	Al-Qassim	Northern Border	Al-Bahah
**Mobility**													
Mild	41,694	31,675	31,626	29,289	34,884	28,237	24,861	36,245	25,735	40,363	33,396	32,928	34,757
Severe	45,286	41,901	39,495	44,662	40,240	42,449	42,414	45,630	37,563	35,338	53,475	56,751	33,614
Extreme	38,785	44,421	40,866	36,994	43,708	49,279	53,651	56,925	57,343	25,694	39,939	47,289	51,186
**Eyesight**													
Mild	26,100	42,746	46,117	40,706	44,467	40,063	46,183	36,579	59,758	35,502	31,789	45,869	32,751
Severe	27,978	28,895	28,924	27,602	34,393	24,352	30,749	25,452	41,028	38,006	19,070	23,755	22,706
Extreme	22,579	31,039	36,823	37,968	33,339	24,574	22,394	25,892	21,666	40,016	30,607	41,236	36,059
**Hearing**													
Mild	14,708	9728	8209	11,449	10,898	8302	11,372	12,283	7703	14,412	10,317	10,571	2718
Severe	14,858	13,005	14,426	8,395	13,266	15,789	9253	14,038	9724	12,701	7784	5993	9878
Extreme	11,596	12,682	13,034	11,836	12,690	12,472	16,802	3381	17,445	10,982	8323	9332	5560
**Speech & Communication**													
Mild	12,577	11,005	10,837	16,944	5715	15,533	11,284	8512	3727	4321	16,232	6926	24,854
Severe	4313	8164	8848	8084	4777	9710	5838	7007	7136	9550	15,198	10,181	18,352
Extreme	20,331	9825	7688	9823	7504	11,553	7153	13,803	3546	21,678	21,131	2144	7195
**Memory**													
Mild	1087	2136	1429		4036	2140	2141	1464	888	4395	6144		4919
Severe	7565	7612	7241	11,257	7324	7259	11,746	6214	4549	4405	4473	1083	15,449
Extreme		1045	502	1128	2759	1533				1632			
**Self-care**													
Mild	3834	2710	1783	1611		5726	4159	4916	2188	1007	2120	3706	
Severe		423	1066			442		1659				2238	
Extreme	6709	988	1087	2252		589							
